# Left Ventricular Ejection Fraction Is Associated with the Risk of Thrombus in the Left Atrial Appendage in Patients with Atrial Fibrillation

**DOI:** 10.1155/2020/3501749

**Published:** 2020-04-24

**Authors:** Beata Uziębło-Życzkowska, Paweł Krzesiński, Agnieszka Jurek, Agnieszka Kapłon-Cieślicka, Iwona Gorczyca, Monika Budnik, Grzegorz Gielerak, Marek Kiliszek, Monika Gawałko, Piotr Scisło, Janusz Kochanowski, Olga Jelonek, Anna Michalska, Katarzyna Starzyk, Krzysztof J. Filipiak, Beata Wożakowska-Kapłon, Grzegorz Opolski

**Affiliations:** ^1^Department of Cardiology and Internal Diseases, Military Institute of Medicine, Warsaw, Poland; ^2^1st Chair and Department of Cardiology, Medical University of Warsaw, Warsaw, Poland; ^3^Clinic of Cardiology and Electrotherapy, Swietokrzyskie Cardiology Centre, Kielce, Poland; ^4^Faculty of Medical and Health Sciences, The Jan Kochanowski University, Kielce, Poland

## Abstract

**Introduction:**

Atrial fibrillation (AF) is associated with high risk of ischemic stroke. The most frequent thrombus location in AF is the left atrial appendage (LAA). Transthoracic echocardiography (TTE) is a basic diagnostic examination in patients (pts) with AF.

**Objectives:**

To analyse the relations between basic echocardiographic features, well-established stroke risk factors, type of AF, and anticoagulation therapy with the incidence of left atrial appendage thrombus (LAAT). *Patients and Methods*. The study group consisted of 768 pts with AF (mean age, 63 years), admitted to three high-reference cardiology departments. Five hundred and twenty-three pts were treated with non-vitamin K antagonist oral anticoagulants (NOACs) and 227 (30%) with vitamin K antagonists (VKAs). The subjects underwent TTE and transesophageal echocardiography (TEE) before cardioversion or ablation.

**Results:**

LAAT was significantly more frequent in pts with reduced left ventricular ejection fraction (LVEF): in 10.6% (7 pts) with LVEF < 40% and in 9.0% (9 pts) with LVEF 40-49%, while only in 5.5% (33 pts) with LVEF > 50%. Compared to pts without LAAT, those with LAAT presented with lower LVEF and higher left atrial diameter (LAD). Multivariate logistic regression revealed the following variables as independent predictors of LAAT: previous bleeding, treatment with VKA, and LVEF.

**Conclusion:**

LAAT is related to lower LVEF and higher LAD. LVEF is one of the independent predictors of LAAT. Even in the case of adequate anticoagulant therapy, it might be prudent to consider TEE before cardioversion or ablation in patients with low LVEF and LA enlargement, especially in the coexistence of other thromboembolic risk factors.

## 1. Introduction

Atrial fibrillation (AF) is the most common sustained cardiac dysrhythmia with stroke as a potential thromboembolic complication. The most frequent thrombus location in AF is the left atrial appendage (LAA). Although risk factors for thromboembolism in AF are well recognized, those strongly related to LAA thrombus (LAAT) formation have not been thoroughly examined. Risk of thromboembolism increases with the duration of arrhythmia because AF predisposes to blood stasis and may lead to LAAT formation. Risk factors such as increased CHA_2_DS_2_-VASc score, structural heart disease, and lower maximal left appendage emptying velocity (LAAV) have been identified as predictors of LAAT [1–4]. Furthermore, reduced left ventricular ejection fraction (LVEF) is a part of the CHA_2_DS_2_-VASc score. However, other echocardiographic risk factors are not so well defined.

Transthoracic echocardiography (TTE) is usually available in patients qualified for cardioversion or ablation. The question as to when transesophageal echocardiography (TEE) should also be performed is still open. The current guidelines [5] state that TEE is not necessary in patients with 3 or more weeks of effective anticoagulation (vitamin K antagonists (VKAs) or non-vitamin K antagonist oral anticoagulants (NOACs)), if they have not presented with cardiac failure or very high thrombotic risk. However, for safety reasons, TEE is performed in a wider group of patients before cardioversion or ablation in everyday practice. We hypothesise that there are some echocardiographic features which can convince us to complete the diagnostics with TEE.

We wanted to determine the actual incidence and predictors of LAAT in AF patients with respect to the TTE result. Thus, we aimed to analyse the relations between basic echocardiographic features, well-established stroke risk factors (included in CHA_2_DS_2_-VASc score), type of AF, and anticoagulation therapy with the incidence of LAAT.

## 2. Patients and Methods

### 2.1. Study Population

We retrospectively evaluated 1858 patients with AF admitted to three high-reference cardiology departments between 2013 and 2017 who underwent transesophageal echocardiography (TEE) before cardioversion or ablation. These patients were included in the database after the patients with valvular AF, history of hemodialysis, and GFR < 15 ml/min/1.73 m^2^ were excluded. Then, from the whole group of 1858 patients, we analysed these patients with reported left ventricular ejection fraction (LVEF) estimated by TTE (this was retrospectively analysed, so not all of the 1858 patients had LVEF reported in the medical base). The final study population included 768 patients. A CHA_2_DS_2_-VASc score was calculated for each study patient as per Lip et al. [6]. HASBLED was also estimated to assess the individual one-year risk of major bleeding [7]. Laboratory tests were performed on fasting peripheral venous blood samples collected in the morning.

### 2.2. Baseline Assessment and Oral Anticoagulation Therapy

All baseline demographic, clinical, laboratory, and echocardiographic data and applied treatment at the time of TEE were retrieved retrospectively from the medical records. Data collected for each patient included age, gender, cardiovascular risk factors, type of AF, and type and duration of oral anticoagulation therapy (OAT). The diagnostic criteria for heart failure (HF) with reduced EF (HFrEF), HFmrEF, and HF with preserved EF (HFpEF) were adopted as recommended in ESC guidelines [8]. Normal LVEF (HFpEF) was typically considered as ≥50% and reduced LVEF (HFrEF) as <40%, and LVEF in the range of 40–49% represented a “grey area,” which was defined as HFmrEF. Paroxysmal AF was defined as lasting ≤7 days [5]. Patients with a history of persistent AF or with paroxysmal and persistent AF were adjudicated as having nonparoxysmal AF.

### 2.3. Transthoracic and Transesophageal Echocardiography

All patients had a standard two-dimensional TTE performed by an experienced echocardiographer as part of their cardiological work-up prior to cardioversion or ablation. LVEF was assessed by modified Simpson's rule in TTE performed during index hospitalization or retrieved from patients' medical records (only exams performed <3 months before index hospitalization were included). Echocardiographic data which were collected retrospectively included left atrial anteroposterior diameter (LAD), left ventricular end diastolic diameter (LVDd) (both measured in the parasternal long-axis view in the late diastole of the left atrium and the left ventricle), and LVEF (calculated according to Simpson's formula employing a 2-dimensional image of the left ventricle (LV) chamber during systole and diastole in the 4- and 2-chamber apical views). Left atrium (LA) enlargement was defined as a LA diameter exceeding >4.0 cm in males and >3.8 cm in females [9]. LV enlargement was defined as a LV end diastolic diameter > 5.8 cm in males and >5.2 cm in females [9]. TEEs were performed in all study patients within 48 hours before the scheduled procedure. In most cases, TEE was made a few hours before cardioversion or ablation. Imaging was performed in a certified (with grade C accreditation from the Section of Echocardiography of the Polish Cardiac Society) echocardiography laboratory using a General Electric Vivid 7 or E95 ultrasound system (General Electric, Milwaukee, Wisconsin) and Philips EPIQ 7 or iE33 ultrasound system (Philips Medical Systems, Andover, Massachusetts, United States). Standard TEE acquisitions, including a continuous sweep from 0 to 180 degrees with short- and long-axes, were performed with focused imaging of the LAA and both of the atria. The study evaluated the presence of a thrombus in LAA and spontaneous echocardiographic contrast (SEC) in both LA and LAA. An LA thrombus was identified as a circular or irregular echodense mass in LA or LAA, not being a part of the endocardium or pectinate muscles [10, 11]. All thrombi were found in the left atrial appendage, and they were described as LAAT. SEC was defined as a smoke-like echo with a characteristic motion during the cardiac cycle [12]. Peak LAA emptying velocity was measured via pulse wave Doppler positioned 1 cm into the orifice of the appendage [13]. LAA velocity was assessed as the mean value during several beats in the patients with AF during TEE.

Emptying LAA velocities of less than 20 cm/s were regarded as decreased [14]. If a thrombus was suspected, all images were reviewed by a second experienced echocardiographer trained in TEE, the cardioversion or ablation procedure was not performed, and a repeat TEE was carried out after a further period of anticoagulation.

Written informed consent for TEE was obtained from all patients.

### 2.4. Statistical Analysis

The statistical analysis was performed using Statistica 12.0 (StatSoft, Inc., Tulsa, OK, USA). The distribution and normality of the data were assessed by visual inspection and the Kolmogorov-Smirnov test. Continuous variables were presented as means ± standard deviations (SD) and categorical variables as absolute and relative frequencies (percentages). To analyse the differences between subgroups of patients with and without LAAT, the Student *t*-test for normally distributed data and the Mann-Whitney *U*-test if the data were not normally distributed were applied. For categorical variables, the chi-square test and the Fisher exact test were used. The discriminatory variables (heart failure, paroxysmal AF, female sex, age > 65 y, bleeding in anamnesis, treatment with VKAs, LA enlargement and LAD (per one cm), LVEF < 50% and LVEF (per one %), and eGFR < 60 ml/min/1.73 m^2^) were then analysed by univariate and multivariate logistic regression to identify independent predictors of LAAT. A *p* value of <0.05 was taken to indicate statistical significance.

## 3. Results

Baseline characteristics of the study population are summarized in Table 1. The majority of patients (*n* = 602, 78%) had normal LVEF. Echocardiographic findings of the study population are summarized in Table 2. Five hundred and twenty-three patients (68%) were treated with NOACs, 227 (30%) with VKAs, and 11 (1.5%) with heparin and 7 patients were without any prior oral anticoagulation. In the group treated with NOACs, 240 (46%) patients received dabigatran, 279 (53%) rivaroxaban, and 4 (0.7%) apixaban. Two hundred and eighty-seven (37%) patients presented with paroxysmal AF.

There were no significant differences between VKAs and NOACs patients with respect to the following: mean age (62.9 ± 9.6 vs. 63.5 ± 11.1 years, ns), hemoglobin (14.1 ± 1.4 vs. 14.1 ± 1.6 g/dl, ns), creatinine (1.08 ± 0.46 vs. 1.04 ± 0.23 mg/d, ns), and eGFR < 60 ml/min/1.73 m^2^ (27.5 vs. 23.8%, ns), through the prevalence of diabetes (26.4 vs. 22.4%, ns), coronary disease (63.0 vs. 68.6%, ns), and stroke (5.3 vs 7.3%, ns) in medical history. However, more VKAs patients presented LVEF < 50% (27.3 vs. 19.3%, *p* = 0.015) and symptoms of HF (40.1 vs. 27.6%, *p* = 0.0007).

The occurrence of LAAT was confirmed in 9.0% (*n* = 9) of patients with LVEF 40-49% and in 10.6% (7) of patients with EF < 40% while only in 5.5% (33) of patients with EF > 50%.

Compared to patients without LAAT, those with LAAT presented with lower LVEF (*p* < 0.0001) and higher LAD (*p* = 0.009). The patients with LAAT presented also with more advanced age (*p* = 0.01) and higher CHA_2_DS_2_-VASc score (*p* = 0.003) (Table 3).

The prevalence of LAAT was higher in the presence of the following: HF (11.7% vs. 4.0%; *p* < 0.0001), age > 65 years (8.5% vs. 4.7%, *p* = 0.004), bleeding in anamnesis (16.0% vs. 5.7%, *p* = 0.004), treatment with VKA (9.7% vs. 5.0%, *p* = 0.01), and LA enlargement (97.1% vs. 81.0%, *p* = 0.01), and lower in the case of paroxysmal AF (in comparison to nonparoxysmal) (2.1% vs. 8.9%, *p* < 0.001) and treatment with NOAC (4.8% vs. 9.8%, *p* = 0.008). The trend for more frequent LAAT in cases with estimated glomerular filtration rate (eGFR) < 60 ml/min (9.0% vs. 5.4%, *p* = 0.08) and LVEF < 50% (9.6% vs. 5.5%, *p* = 0.05) was also noted.

There were no statistically significant differences for the following: age > 75 years (5.3% vs. 6.5%), hypertension (6.4% vs. 6.4%), diabetes mellitus (8.9% vs. 5.6%), stroke/transient ischemic attack (1.9% vs. 6.7%), vascular disease (1.9% vs. 6.7%), female sex (7.7% vs. 5.5%), renal abnormal function (8.0% vs. 6.2%), liver abnormal function (6.4% vs. 6.4%), reduced NOAC (5.6% vs. 6.4%), heparin (9.1% vs. 8.1%), and LV enlargement (9.0% vs. 5.8%).

Multivariate logistic regression revealed the following variables as independent predictors of LAAT: previous bleeding (HR = 3.46; 95% CI: 1.18-10.16; *p* = 0.02), treatment with VKA (OR = 2.84; 95% CI: 1.17-6.88; *p* = 0.02), and LVEF (OR = 0.89; 95% CI: 0.83-0.96; *p* = 0.001) (Table 4). It means that the relative risk of LAAT increases 110% per every reduction of LVEF by 10%.

## 4. Discussion

In our analysis, left ventricular systolic dysfunction expressed as decreased LVEF was revealed to be a powerful and independent predictor of LAAT formation in AF patients. Several studies assessed the incidence of LAAT depending on LVEF. According to the study of Khan et al. [3], LAAT is not observed in paroxysmal AF patients with normal LVEF. Another large study (*n* = 1010) showed that moderate to severe LV systolic dysfunction (shown via 2-dimensional echocardiography) was a strong predictor of stroke (relative risk, 2.5; *p* < 0.001) [15].

In our study, the occurrence of LAAT was significantly more frequent in patients with LVEF < 50%, but it should be noted that LAAT was found even in patients with normal LVEF. Therefore, it appears that methods combining clinical and echocardiographic findings could be very useful in making a decision about the TEE performance before cardioversion or ablation. Providência et al. [16] based on predictors of LAAT (CRP, atrial volume, troponin, episode duration, and stroke or embolism in anamnesis) proposed the CATES score, which enabled to predict AF patients with very low risk of thromboembolic events.

The same authors [17] tried to evaluate and compare the accuracy of the CHA_2_DS_2_-VASc scale in the prediction of LAAT and test the additive value of TTE-derived parameters as a possible refinement for these classifications. No significant correlations were found between CHA_2_DS_2_-VASc and the occurrence of LAAT, dense SEC, and LAAV. In contrast, in our population, twice as numerous as in the above mentioned study, patients with LAAT had a higher CHA_2_DS_2_-VASc score. Similarly, the significant value of the CHA_2_DS_2_-VASc score was demonstrated by other authors. In the study of Frenkel et al. [18], no patient with a CHA_2_DS_2_-VASc score of 0 and normal LVEF (in this study described as ≥55%) had LAAT.

Among the echocardiographic parameters that showed a significant value in predicting LAAT in our study, LA enlargement should be commented on. Previous studies identified LA enlargement as a significant independent predictor of LAAT [2, 3, 18–20]. These studies, similar to ours, suggested that LA enlargement was predictive of thromboembolic findings in patients with nonvalvular AF. The indexed and nonindexed LA anterior-posterior diameter and indexed LA ellipsoid volume in the study of Radwan [19] were the most accurate parameters for predicting thromboembolic findings. These results can be explained by observations of Iwakura et al. [21], who described that decreased LVEF and elevated LV filling pressure led to LA dilatation and LAAT formation. Similarly, Goldman et al. [14] suggested that chronic elevation of LA pressure led to LA volume and pressure overload and the deterioration of LAA contractility, resulting in blood stasis and thrombus formation. The Stroke Prevention in Atrial Fibrillation (SPAF-III) study evaluated that patients' clinical characteristics, TEE findings, and subsequent cardioembolic events were correlated with the LAA flow velocity in multivariate analysis. Herring et al. [22] demonstrated that in patients on uninterrupted warfarin therapy LAD > 4.6 cm as well as CHA_2_DS_2_-VAScscore ≥ 1, they could identify 91.5% of those at risk of developing LAAT.

As expected, the prevalence of LAAT was lower in the case of paroxysmal AF (in comparison to nonparoxysmal) and treatment with NOACs. In their latest study, Kapłon-Cieślicka et al. [23] showed that AF type is one of two (with renal dysfunction) variables not included in the CHA_2_DS_2_-VASc score which are independent predictors of LAAT, and they might improve thromboembolic risk stratification. Recently, Alqarawi et al. [24] demonstrated that none of the patients with paroxysmal AF or on NOAC were found to have LAAT. The authors have found only three cases of LAAT in the whole group of 668 patients, and all of them had persistent AF and were on warfarin. Similar results were obtained by Wyrembak et al. [25], who analysed 937 routine pre-AF ablation TEE procedures performed in patients treated with warfarin (*n* = 517) or NOACs (*n* = 420). The incidence of LAAT was higher in patients treated with warfarin (1.55%, 8 of 517) compared with patients treated with NOACs (0.24%, 1 of 420, *p* = 0.047). In contrast, Gawałko et al. [4] have found no difference in the prevalence of LAAT between patients on warfarin and those on NOACs (6.9% vs. 5.5%; *p* = 0.4). In the study by Frenkel et al. [18], after ≥4weeks of therapy, the prevalence of LAAT among patients on NOACs was comparable to that among patients on warfarin.

### 4.1. Clinical Implications

Our results might be helpful in making a decision about TEE performance before cardioversion or ablation. Some findings in TTE (low LVEF and large LA) might suggest performing TEE even though no standard recommendation is fulfilled. More advanced LV and LA dysfunction should also convince us of more aggressive anticoagulation in such group of patients.

### 4.2. Study Limitations

Our study has several limitations, including primarily its retrospective design. For this reason, we had to evaluate these echocardiographic and other parameters, which were identified in all our AF patients. This creates obvious limitations in the study, such as the fact that we had to analyse the anteroposterior LA dimension to determine its enlargement. We are aware that the LAV/LAVI would be a better parameter for assessing the LA abnormalities. TEE examinations were performed by three different echocardiographers. However, when a thrombus was suspected, all images were reviewed by a second experienced echocardiographer trained in TEE. We are also aware that TEE does not have 100% sensitivity to detect LAAT. However, it has been the gold standard so far. Next, our study hypothesis is based on the surrogate TEE marker endpoint, so it should be clinically validated in a study involving a thromboembolic event as the primary endpoint. Nonetheless, it is known that the main location of thrombi leading to stroke in AF patients is the left atrial appendage. Finally, we noted that VKAs were preferred in patients with lower LVEF, but it did not influence the results of multivariate logistic regression, where treatment with VKA was revealed to be independent from both those variables.

## 5. Conclusion

Left atrial appendage thrombus is significantly more common in patients with reduced LVEF and LA enlargement. LVEF is one of the independent predictors of LAAT. Even in the case of adequate anticoagulant therapy, it might be prudent to consider TEE before cardioversion or ablation in patients with low LVEF and LA enlargement, especially with the coexistence of other thromboembolic risk factors.

## Figures and Tables

**Table 1:** Baseline characteristics of the study population.

Parameter^number of patients^	
*Demographic data*	
Age^768^ (years, mean (SD))	63.36 (10.71) [17-90]
Age ≥ 75 (years)	95 (12%)
Age 65-74 (years)	271 (35%)
Age > 65 (years)	340 (44%)
BMI^567^ (kg/m^2^, mean (SD))	29.46 (5.38)
Female: *n* (%)	297 (39%)
*Type of procedure^768^:n(%)*	
Ablation	365 (47%)
Cardioversion	403 (53%)
*Type of AF^768^:n(%)*	
AF paroxysmal	287 (37%)
*Clinical data:n(%)*	
HF^766^	240 (31%)
NYHA I-II	209 (87.1%)
NYHA III	26 (10.8%)
NYHA IV	5 (2.1%)
HT^768^	581 (75%)
DM^767^	179 (23%)
Stroke^768^	52 (6.8%)
Coronary disease^768^	506 (66%)
Biological aortic prosthesis^768^	19 (2.5%)
CHA_2_DS_2_-VASC score^768^ (mean (SD))	2.48 (1.53)
CHA_2_DS_2_‐VASC = 0: *n* (%)	65 (8.6%)
CHA_2_DS_2_‐VASC = 1: *n* (%)	154 (20%)
CHA_2_DS_2_‐VASC ≥ 2: *n* (%)	549 (71.4%)
HASBLED score^768^ (mean (SD))	1.58 (0.99)
Liver abnormal function^767^	110 (14%)
Renal abnormal function^767^	100 (13%)
Bleeding in anamnesis^768^	50 (6.5%)
*Laboratory data (mean (SD))^761^*	
Hemoglobin (g/dl)	14.10 (1.54)
Creatinine (mg/dl)	1.04 (0.31)
EGFR < 60 ml/min/1.73 m^2^	189 (25%)
*Type of anticoagulation therapy^768^:n(%)*	
NOAC	523 (68%)
(i) Dabigatran	240 (46%)
(ii) Rivaroxaban	279 (53%)
(iii) Apixaban	4 (0.7%)
VKA	227 (30%)
OAC	750 (98%)
Reduced NOAC	54 (7%)
Heparin	11 (1.5%)

Abbreviations: AF: atrial fibrillation; BMI: body mass index; DM: diabetes mellitus; eGFR: estimated glomerular filtration rate; HF: heart failure; HT: hypertension; NOAC: non-vitamin K antagonist oral anticoagulants; OAC: oral anticoagulation therapy; VKA: vitamin K antagonist.

**Table 2:** Echocardiographic findings of the study population.

Echocardiographic parameter	
LVEF^768^ (%, mean (SD))	54.47 (10.08)
LVEF < 50%^768^ (*n* (%))	166 (22%)
LAD^543^ (cm, mean (SD))	4.49 (0.55)
LA enlargement^545^: *n* (%)	447 (82%)
LVDd^513^ (cm, mean (SD))	5.16 (0.64)
LV enlargement^515^: *n* (%)	100 (19%)
LAAT^768^: *n* (%)	49 (6.4%)
SEC LA^584^: *n* (%)	146 (25%)
LAAV^550^ (cm/s, mean (SD))	42 (21)

Abbreviations: LA: left atrial; LAA: left atrial appendage; LAAV: left atrial appendage emptying velocity; LAAT: thrombus in left atrial; LAD: left atrium diameter; LVDd: left ventricular diastolic diameter; LVEF: left ventricular ejection fraction; SEC LA: spontaneous echocardiographic contrast in the left atrial.

**Table 3:** Comparison of baseline characteristics between patients with (LAAT (+)) and without (LAAT (−)) left atrial thrombus.

	LAAT (−)	LAAT (+)	*p*
Age (years, mean (SD))	63.1 (8.1)	67.2 (8.1)	0.01
*Laboratory data (mean (SD))*			
Hemoglobin (mg/dl)	14.11 (1.56)	13.86 (1.31)	0.23
Creatinine (mg/dl)	1.04 (0.32)	1.07 (0.24)	0.41
eGFR (ml/min/1.73 m^2^)	76.7 (18.2)	71.4 (19.5)	0.054
AST (U/l)	27.81 (19.20)	28.76 (13.80)	0.78
ALT (U/l)	32.16 (19.42)	29.65 (16.85)	0.49
INR	1.68 (0.87)	1.84 (0.73)	0.04
APTT (s)	38.01 (15.53)	41.29 (14.79)	0.16
*Echocardiographic parameter (mean (SD))*			
LVEF (%)	54.8 (9.9)	49.5 (12.1)	<0.0001
LAAV (cm/s)	42 (21)	35 (28)	0.004
LAD (cm)	4.47 (0.55)	4.77 (0.59)	0.009
LVDd (cm)	5.15 (0.62)	5.18 (0.89)	0.83
*Scales of risk*			
CHA_2_DS_2_-VAScscore (points)	2.44 (1.53)	3.08 (1.33)	0.002
HASBLED score (points)	1.57 (0.99)	1.83 (1.03)	0.10

Abbreviations: ALT: alanine aminotransferase; AST: aspartate aminotransferase; INR: international normalized ratio; APTT: activated partial thromboplastin time. Other abbreviations are defined the same as in Tables 1 and 2.

**Table 4:** The results of univariate and multivariate logistic regression.

Variable	Univariate		Multivariate	
	OR (95% CI)	*p*	OR (95% CI)	*p*
Heart failure	2.97 (1.43-6.17)	0.003	1.62 (0.65-4.05)	0.3
Paroxysmal AF	0.28 (0.11-0.68)	0.005	0.41 (0.12-1.37)	0.31
Female	2.06 (1.01-4.24)	0.05	1.99 (0.74-5.32)	0.17
Age > 65 y	2.91 (1.36-6.24)	0.006	2.50 (0.92-6.77)	0.07
eGFR < 60 ml/min/1.73 m^2^	2.38 (1.15-4.91)	0.019	0.93 (0.36-2.41)	0.87
Bleeding	3.61 (1.39-9.37)	0.008	3.46 (1.18-10.16)	0.02
Treatment with VKA	2.65 (1.31-5.39)	0.007	2.84 (1.17-6.88)	0.02
LAD (per one cm)	2.18 (1.05-4.58)	0.04	1.64 (0.75-3.58)	0.21
LVEF (per one %)	0.86 (0.80-0.94)	0.0004	0.89 (0.83-0.96)	0.001

Abbreviations are defined the same as in Tables 1 and 2.

## Data Availability

The data used to support the findings of this study are available from the corresponding author upon request.

## References

[1] Scherr D., Dalal D., Chilukuri K. (2009). Incidence and predictors of left atrial thrombus prior to catheter ablation of atrial fibrillation. *Journal of Cardiovascular Electrophysiology*.

[2] Puwanant S., Varr B. C., Shrestha K. (2009). Role of the CHADS2 score in the evaluation of thromboembolic risk in patients with atrial fibrillation undergoing transesophageal echocardiography before pulmonary vein isolation. *Journal of the American College of Cardiology*.

[3] Khan M. N., Usmani A., Noor S. (2008). Low incidence of left atrial or left atrial appendage thrombus in patients with paroxysmal atrial fibrillation and normal EF who present for pulmonary vein antrum isolation procedure. *Journal of Cardiovascular Electrophysiology*.

[4] Gawałko M., Kapłon-Cieślicka A., Budnik M. (2017). Comparison of different oral anticoagulant regimens in patients with atrial fibrillation undergoing ablation or cardioversion. *Pol Arch Intern Med*.

[5] Kirchhof P., Benussi S., Kotecha D. (2016). 2016 ESC guidelines for the management of atrial fibrillation developed in collaboration with EACTS. *European Heart Journal*.

[6] Lip G. Y., Nieuwlaat R., Pisters R., Lane D. A., Crijns H. J. (2010). Refining clinical risk stratification for predicting stroke and thromboembolism in atrial fibrillation using a novel risk factor-based approach: the Euro Heart Survey on atrial fibrillation. *Chest*.

[7] Pisters R., Lane D. A., Nieuwlaat R., de Vos C. B., Crijns H. J. G. M., Lip G. Y. H. (2010). A novel user-friendly score (HAS-BLED) to assess 1-year risk of major bleeding in patients with atrial fibrillation: the Euro Heart Survey. *Chest Journal*.

[8] Ponikowski P., Voors A. A., Anker S. D. (2016). 2016 ESC guidelines for the diagnosis and treatment of acute and chronic heart failure. *European Heart Journal*.

[9] Lang R. M., Badano L. P., Mor-Avi V. (2015). Recommendations for cardiac chamber quantification by echocardiography in adults: an update from the American Society of Echocardiography and the European Association of Cardiovascular Imaging. *Journal of the American Society of Echocardiography*.

[10] Manning W. J., Weintraub R. M., Waksmonski C. A. (1995). Accuracy of Transesophageal Echocardiography for Identifying Left Atrial Thrombi: A Prospective, Intraoperative Study. *Annals of Internal Medicine*.

[11] Aschenberg W., Schlüter M., Kremer P., Schröder E., Siglow V., Bleifeld W. (1986). Transesophageal two-dimensional echocardiography for the detection of left atrial appendage thrombus. *Journal of the American College of Cardiology*.

[12] Fatkin D., Kelly R. P., Feneley M. P. (1994). Relations between left atrial appendage blood flow velocity, spontaneous echocardiographic contrast and thromboembolic risk in vivo. *Journal of the American College of Cardiology*.

[13] Goldberg Y. H., Gordon S. C., Spevack D. M., Gordon G. M. (2010). Disparities in emptying velocity within the left atrial appendage. *European Journal of Echocardiography*.

[14] Goldman M. E., Pearce L. A., Hart R. G. (1999). Pathophysiologic correlates of thromboembolism in nonvalvular atrial fibrillation: I. Reduced flow velocity in the left atrial appendage (the Stroke Prevention in Atrial Fibrillation [SPAF-III] study). *Journal of the American Society of Echocardiography*.

[15] (1998). Echocardiographic predictors of stroke in patients with atrial Fibrillation. *Archives of Internal Medicine*.

[16] Providência R., Faustino A., Paiva L. (2012). Cardioversion safety in patients with nonvalvular atrial fibrillation: which patients can be spared transesophageal echocardiography?. *Blood Coagulation & Fibrinolysis*.

[17] Providência R., Botelho A., Trigo J. (2012). Possible refinement of clinical thromboembolism assessment in patients with atrial fibrillation using echocardiographic parameters. *Europace*.

[18] Frenkel D., D’Amato S. A., Al-Kazaz M. (2016). Prevalence of Left Atrial Thrombus Detection by Transesophageal Echocardiography: A Comparison of Continuous Non–Vitamin K Antagonist Oral Anticoagulant Versus Warfarin Therapy in Patients Undergoing Catheter Ablation for Atrial Fibrillation. *JACC: Clinical Electrophysiology*.

[19] Radwan H. I. (2017). Relation between left atrial measurements and thromboembolic risk markers assessed by echocardiography in patients with nonvalvular atrial fibrillation: a cross-sectional study. *Egypt Heart J*.

[20] Calvo N., Mont L., Vidal B. (2011). Usefulness of transoesophageal echocardiography before circumferential pulmonary vein ablation in patients with atrial fibrillation: is it really mandatory?. *Europace*.

[21] Iwakura K., Okamura A., Koyama Y. (2011). Effect of elevated left ventricular diastolic filling pressure on the frequency of left atrial appendage thrombus in patients with nonvalvular atrial fibrillation. *The American Journal of Cardiology*.

[22] Herring N., Page S. P., Ahmed M. (2013). The prevalence of low left atrial appendage emptying velocity and thrombus in patients undergoing catheter ablation for atrial fibrillation on uninterrupted peri-procedural warfarin therapy. *J Atr Fibrillation*.

[23] Kapłon-Cieślicka A., Budnik M., Gawałko M. (2019). Atrial fibrillation type and renal dysfunction as important predictors of left atrial thrombus. *Heart*.

[24] Alqarawi W., Birnie D. H., Spence S. (2019). Prevalence of left atrial appendage thrombus detected by transoesophageal echocardiography before catheter ablation of atrial fibrillation in patients anticoagulated with non-vitamin K antagonist oral anticoagulants. *Europace*.

[25] Wyrembak J., Campbell K. B., Steinberg B. A. (2017). Incidence and predictors of left atrial appendage thrombus in patients treated with nonvitamin K oral anticoagulants versus warfarin before catheter ablation for atrial fibrillation. *The American Journal of Cardiology*.

